# Role of ApoE polymorphism in Dementia

**DOI:** 10.1002/alz.087829

**Published:** 2025-01-09

**Authors:** Rachna Agarwal, Suman Kushwaha, Chandra Bhushan Tripathi

**Affiliations:** ^1^ Institute of Human Behavior & Allied Sciences, New Delhi, New Delhi India; ^2^ IHBAS, New Delhi, New Delhi, Delhi India; ^3^ Institute of Human Behaviour and Allied Sciences, Delhi India

## Abstract

**Background:**

ApoE polymorphism especially APOE ε4 play a central role in AD pathophysiology through Aβ‐dependent and Aβ‐independent neuropathogenic pathway in the Alzheimer’s disease.

**Method:**

A cross‐sectional study was performed on non diseased and diseased subjects with stroke from outpatient services of Neurology department of Institute of Human Behavior & Allied Sciences (IHBAS), New Delhi (India). Subjects diagnosed with various dementias including Alzheimer’s disease, non AD dementias and dementia with Behavior & Psychological symptoms were taken. APOE genotyping was done in all subjects by ARMS‐PCR method.

**Result:**

The most common allele APOE ε3ε3 was observed across all dementia groups (AD, non AD dementia. Dementia with BPSD & forgetfulness, whereas ε2ε4 was least commonly observed. Multivariable logistic regression analysis showed that the presence of APOE ε4 genotyping is significantly increasing the risk of AD three times more as compared to other alleles (Adjusted Odds Ratio: 3.07, 95% CI: 1.39 ‐ 6.78). The age and gender did not emerge out as significant contributors in AD. In group of AD with BPSD, the most common Behavior & Psychological symptoms observed was paranoid & delusional ideation.

**Conclusion:**

APOE ε4 is a risk factor for Alzheimer's disease.

